# Bifenox: methyl 5-(2,4-di­chloro­phen­oxy)-2-nitro­benzoate

**DOI:** 10.1107/S1600536813018266

**Published:** 2013-07-06

**Authors:** Youngeun Jeon, Jineun Kim, Seonghwa Cho, Tae Ho Kim

**Affiliations:** a Department of Chemistry and Research Institute of Natural Sciences, Gyeongsang National University, Jinju 660-701, Republic of Korea

## Abstract

In the title compound, the herbicide bifenox, C_14_H_9_Cl_2_NO_5_, the dihedral angle between the dichlorobenzene and nitro­benzene rings is 78.79 (14)°. In the crystal, C—H⋯O hydrogen bonds give rise to a three-dimensional network structure in which there are both a π–π inter­action [ring centroid separation = 3.6212 (16) Å] and a C—Cl⋯π inter­action [Cl⋯ring centroid = 3.4754 (8) Å]. In addition, short Cl⋯Cl contacts [3.3767 (11) and 3.3946 (11) Å] are present.

## Related literature

For information on the insecticidal activity of the title compound, see: Jinno *et al.* (1999 ▶); O’Neil (2001 ▶). For a related crystal structure, see: Smith *et al.* (1981 ▶).
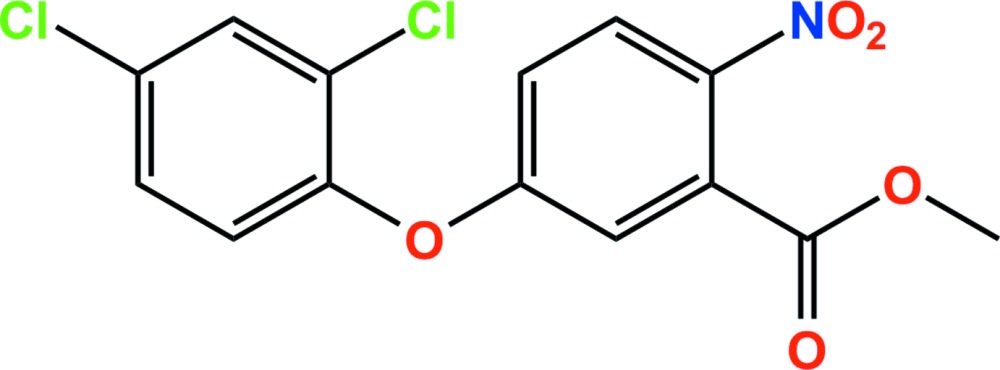


## Experimental

### 

#### Crystal data

C_14_H_9_Cl_2_NO_5_
*M* *_r_* = 342.12
Triclinic, 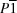

*a* = 8.4945 (7) Å
*b* = 9.9610 (8) Å
*c* = 10.3969 (8) Å
α = 64.601 (1)°
β = 68.720 (1)°
γ = 70.421 (1)°
*V* = 723.48 (10) Å^3^
*Z* = 2
Mo *K*α radiation
μ = 0.47 mm^−1^
*T* = 173 K
0.20 × 0.10 × 0.10 mm

#### Data collection

Bruker APEXII CCD-detector diffractometer
Absorption correction: multi-scan (*SADABS*; Bruker, 2006 ▶) *T*_min_ = 0.912, *T*_max_ = 0.954
5707 measured reflections
2830 independent reflections
2217 reflections with *I* > 2σ(*I*)
*R*_int_ = 0.029

#### Refinement

*R*[*F* ^2^ > 2σ(*F* ^2^)] = 0.047
*wR*(*F* ^2^) = 0.098
*S* = 1.06
2830 reflections
200 parameters
H-atom parameters constrained
Δρ_max_ = 0.29 e Å^−3^
Δρ_min_ = −0.33 e Å^−3^

### 

Data collection: *APEX2* (Bruker, 2006 ▶); cell refinement: *SAINT* (Bruker, 2006 ▶); data reduction: *SAINT*; program(s) used to solve structure: *SHELXS97* (Sheldrick, 2008 ▶); program(s) used to refine structure: *SHELXL97* (Sheldrick, 2008 ▶); molecular graphics: *SHELXTL* (Sheldrick, 2008 ▶); software used to prepare material for publication: *SHELXTL*.

## Supplementary Material

Crystal structure: contains datablock(s) global, I. DOI: 10.1107/S1600536813018266/zs2269sup1.cif

Structure factors: contains datablock(s) I. DOI: 10.1107/S1600536813018266/zs2269Isup2.hkl

Supplementary material file. DOI: 10.1107/S1600536813018266/zs2269Isup3.cml

Additional supplementary materials:  crystallographic information; 3D view; checkCIF report

## Figures and Tables

**Table 1:** Hydrogen-bond geometry (Å, °)

*D*—H⋯*A*	*D*—H	H⋯*A*	*D*⋯*A*	*D*—H⋯*A*
C5—H5⋯O4^i^	0.95	2.41	3.290 (3)	153
C6—H6⋯O5^ii^	0.95	2.43	3.243 (3)	143
C8—H8⋯O2^iii^	0.95	2.58	3.435 (3)	151

Symmetry codes: (i) 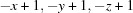; (ii) 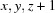; (iii) 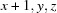.

**Fig. 1.**
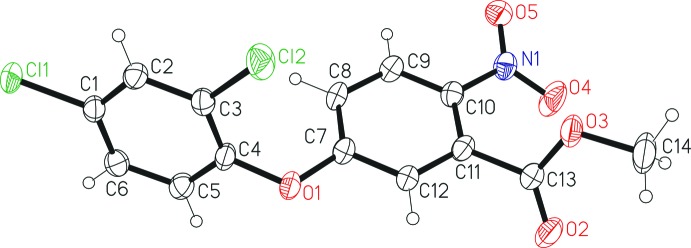
The molecular structure of the title compound showing the atom numbering scheme. Displacement ellipsoids are drawn at the 50% probability level. H atoms are shown as small spheres of arbitrary radius.

**Fig. 2.**
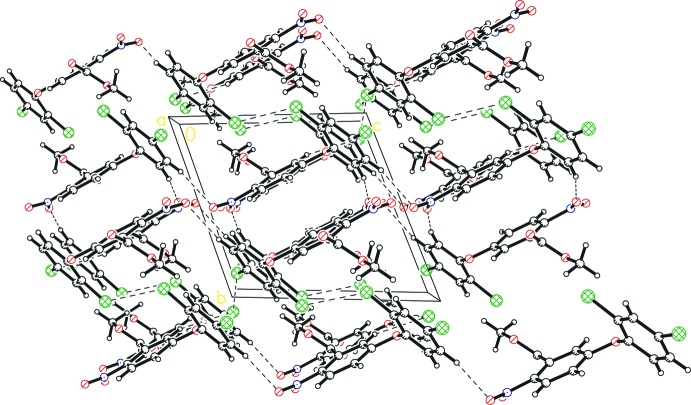
Crystal packing of the title compound with weak intermolecular Cl···Cl interactions and C—H···O hydrogen bonds shown as dashed lines.

## supplementary crystallographic information

### Comment

The title compound bifenox, C_14_H_9_Cl_2_NO_5_, is commonly used as a photobleaching herbicide for the control of many types of weeds (Jinno *et al.*, 1999; O'Neil, 2001) and its crystal structure is reported herein. In this compound (Fig. 1), the dihedral angle between the dichlorophenyl ring and the nitrobenzene ring is 78.79 (14)°. All bond lengths and bond angles are normal and comparable to those observed in a similar phenoxy herbicide structure, methyl 2-[4-(2,4-dichlorophenoxy)phenoxy]propionate (diclofop methyl) (Smith *et al.*, 1981).

In the crystal structure (Fig. 2), intermolecular C—H···O hydrogen bonds are observed (Table 1), giving a three-dimensional network structure (Fig. 2). In this structure there are both a π–π interaction between the dichlorophenyl rings [ring centroid separation = 3.6212 (16) Å] and a C3—Cl2···π interaction with the nitrobenzene ring [Cl2···*Cg*^iv^ = 3.4754 (8) Å]. In addition, short Cl···Cl contacts [Cl1···Cl1^v^, 3.3767 (11) Å and Cl2···Cl2^iv^3.3946 (11) Å] are present [for symmetry codes: (iv), *-x*+2, *-y*, *-z*+1 and (v), *-x*+3, *-y*, *-z*+2].

### Experimental

The title compound was purchased from Wako Pure Chemicals. Slow evaporation of a solution in CH_2_Cl_2_ gave single crystals suitable for X-ray analysis.

### Refinement

All H-atoms were positioned geometrically and refined using a riding model with d(C—H) = 0.95 Å, *U*_iso_ = 1.2*U*_eq_(C) for C*sp*^2^—H and d(C—H) = 0.98 Å, *U*_iso_ = 1.5*U*_eq_(C) for CH_3_ groups.

### Crystal data

**Table:**

C_14_H_9_Cl_2_NO_5_	*Z* = 2
*M**_r_* = 342.12	*F*(000) = 348
Triclinic, *P*1	*D*_x_ = 1.570 Mg m^−^^3^
Hall symbol: -P 1	Melting point = 357–359 K
*a* = 8.4945 (7) Å	Mo *K*α radiation, λ = 0.71073 Å
*b* = 9.9610 (8) Å	Cell parameters from 2834 reflections
*c* = 10.3969 (8) Å	θ = 2.3–27.4°
α = 64.601 (1)°	µ = 0.47 mm^−^^1^
β = 68.720 (1)°	*T* = 173 K
γ = 70.421 (1)°	Block, colourless
*V* = 723.48 (10) Å^3^	0.20 × 0.10 × 0.10 mm

### Data collection

**Table:**

Bruker APEXII CCD-detector diffractometer	2830 independent reflections
Radiation source: fine-focus sealed tube	2217 reflections with *I* > 2σ(*I*)
Graphite monochromator	*R*_int_ = 0.029
φ and ω scans	θ_max_ = 26.0°, θ_min_ = 2.2°
Absorption correction: multi-scan (*SADABS*; Bruker, 2006)	*h* = −10→10
*T*_min_ = 0.912, *T*_max_ = 0.954	*k* = −12→12
5707 measured reflections	*l* = −12→12

### Refinement

**Table:**

Refinement on *F*^2^	Primary atom site location: structure-invariant direct methods
Least-squares matrix: full	Secondary atom site location: difference Fourier map
*R*[*F*^2^ > 2σ(*F*^2^)] = 0.047	Hydrogen site location: inferred from neighbouring sites
*wR*(*F*^2^) = 0.098	H-atom parameters constrained
*S* = 1.06	*w* = 1/[σ^2^(*F*_o_^2^) + (0.0274*P*)^2^ + 0.5552*P*] where *P* = (*F*_o_^2^ + 2*F*_c_^2^)/3
2830 reflections	(Δ/σ)_max_ < 0.001
200 parameters	Δρ_max_ = 0.29 e Å^−^^3^
0 restraints	Δρ_min_ = −0.33 e Å^−^^3^

### Special details

**Table:**

Geometry. All e.s.d.'s (except the e.s.d. in the dihedral angle between two l.s. planes) are estimated using the full covariance matrix. The cell e.s.d.'s are taken into account individually in the estimation of e.s.d.'s in distances, angles and torsion angles; correlations between e.s.d.'s in cell parameters are only used when they are defined by crystal symmetry. An approximate (isotropic) treatment of cell e.s.d.'s is used for estimating e.s.d.'s involving l.s. planes.
Refinement. Refinement of *F*^2^ against ALL reflections. The weighted *R*-factor *wR* and goodness of fit *S* are based on *F*^2^, conventional *R*-factors *R* are based on *F*, with *F* set to zero for negative *F*^2^. The threshold expression of *F*^2^ > σ(*F*^2^) is used only for calculating *R*-factors(gt) *etc*. and is not relevant to the choice of reflections for refinement. *R*-factors based on *F*^2^ are statistically about twice as large as those based on *F*, and *R*- factors based on ALL data will be even larger.

### Fractional atomic coordinates and isotropic or equivalent isotropic displacement parameters (Å^2^)

**Table:**

	*x*	*y*	*z*	*U*_iso_*/*U*_eq_	
Cl1	1.32195 (8)	0.11710 (8)	0.94482 (7)	0.03459 (19)	
Cl2	1.02470 (9)	−0.03963 (9)	0.66832 (8)	0.0410 (2)	
O1	0.7224 (2)	0.2017 (2)	0.74018 (19)	0.0329 (5)	
O2	0.2690 (2)	0.3461 (3)	0.4414 (2)	0.0493 (6)	
O3	0.4770 (2)	0.2201 (2)	0.3023 (2)	0.0379 (5)	
O4	0.5339 (3)	0.5178 (3)	0.1364 (2)	0.0515 (6)	
O5	0.8081 (2)	0.5119 (2)	0.0585 (2)	0.0371 (5)	
N1	0.6782 (3)	0.4819 (2)	0.1575 (2)	0.0301 (5)	
C1	1.1456 (3)	0.1440 (3)	0.8819 (3)	0.0256 (6)	
C2	1.1569 (3)	0.0548 (3)	0.8061 (3)	0.0266 (6)	
H2	1.2589	−0.0193	0.7880	0.032*	
C3	1.0159 (3)	0.0761 (3)	0.7571 (3)	0.0256 (6)	
C4	0.8694 (3)	0.1865 (3)	0.7802 (3)	0.0267 (6)	
C5	0.8603 (3)	0.2739 (3)	0.8570 (3)	0.0285 (6)	
H5	0.7588	0.3487	0.8742	0.034*	
C6	0.9992 (3)	0.2526 (3)	0.9092 (3)	0.0281 (6)	
H6	0.9935	0.3117	0.9627	0.034*	
C7	0.7221 (3)	0.2709 (3)	0.5937 (3)	0.0264 (6)	
C8	0.8489 (3)	0.3448 (3)	0.4844 (3)	0.0272 (6)	
H8	0.9450	0.3493	0.5076	0.033*	
C9	0.8337 (3)	0.4121 (3)	0.3410 (3)	0.0267 (6)	
H9	0.9191	0.4638	0.2648	0.032*	
C10	0.6932 (3)	0.4035 (3)	0.3092 (3)	0.0244 (5)	
C11	0.5659 (3)	0.3277 (3)	0.4177 (3)	0.0254 (6)	
C12	0.5811 (3)	0.2624 (3)	0.5610 (3)	0.0260 (6)	
H12	0.4951	0.2116	0.6375	0.031*	
C13	0.4188 (3)	0.3035 (3)	0.3881 (3)	0.0273 (6)	
C14	0.3450 (4)	0.1934 (4)	0.2634 (4)	0.0501 (9)	
H14A	0.2658	0.1409	0.3533	0.075*	
H14B	0.3999	0.1304	0.2013	0.075*	
H14C	0.2802	0.2909	0.2093	0.075*	

### Atomic displacement parameters (Å^2^)

**Table:**

	*U*^11^	*U*^22^	*U*^33^	*U*^12^	*U*^13^	*U*^23^
Cl1	0.0281 (3)	0.0478 (4)	0.0343 (4)	−0.0092 (3)	−0.0157 (3)	−0.0130 (3)
Cl2	0.0433 (4)	0.0470 (5)	0.0498 (5)	−0.0054 (3)	−0.0189 (3)	−0.0297 (4)
O1	0.0243 (9)	0.0494 (12)	0.0270 (10)	−0.0116 (9)	−0.0117 (8)	−0.0081 (9)
O2	0.0217 (10)	0.0761 (16)	0.0698 (15)	−0.0060 (10)	−0.0101 (10)	−0.0478 (13)
O3	0.0288 (10)	0.0505 (13)	0.0516 (13)	−0.0017 (9)	−0.0186 (9)	−0.0315 (11)
O4	0.0353 (12)	0.0652 (15)	0.0457 (13)	−0.0094 (11)	−0.0264 (10)	0.0000 (11)
O5	0.0334 (11)	0.0450 (12)	0.0299 (10)	−0.0096 (9)	−0.0056 (9)	−0.0117 (9)
N1	0.0298 (12)	0.0278 (12)	0.0343 (13)	−0.0021 (10)	−0.0161 (10)	−0.0090 (10)
C1	0.0240 (13)	0.0306 (14)	0.0216 (13)	−0.0088 (11)	−0.0088 (10)	−0.0040 (11)
C2	0.0234 (13)	0.0279 (14)	0.0264 (14)	−0.0032 (11)	−0.0087 (11)	−0.0073 (11)
C3	0.0269 (13)	0.0272 (14)	0.0245 (13)	−0.0073 (11)	−0.0082 (11)	−0.0083 (11)
C4	0.0234 (13)	0.0334 (15)	0.0247 (13)	−0.0110 (11)	−0.0108 (10)	−0.0040 (11)
C5	0.0256 (13)	0.0281 (14)	0.0316 (14)	−0.0012 (11)	−0.0099 (11)	−0.0115 (12)
C6	0.0324 (14)	0.0276 (14)	0.0273 (14)	−0.0085 (11)	−0.0081 (11)	−0.0106 (12)
C7	0.0226 (13)	0.0278 (14)	0.0295 (14)	−0.0024 (11)	−0.0123 (11)	−0.0083 (12)
C8	0.0231 (13)	0.0272 (14)	0.0355 (15)	−0.0045 (11)	−0.0151 (11)	−0.0093 (12)
C9	0.0246 (13)	0.0230 (13)	0.0310 (15)	−0.0057 (11)	−0.0106 (11)	−0.0046 (11)
C10	0.0240 (13)	0.0201 (13)	0.0287 (14)	−0.0009 (10)	−0.0115 (11)	−0.0074 (11)
C11	0.0194 (12)	0.0238 (13)	0.0351 (15)	−0.0006 (10)	−0.0104 (11)	−0.0126 (12)
C12	0.0215 (12)	0.0315 (14)	0.0267 (14)	−0.0052 (11)	−0.0083 (11)	−0.0103 (12)
C13	0.0233 (14)	0.0287 (14)	0.0304 (14)	−0.0042 (11)	−0.0098 (11)	−0.0096 (12)
C14	0.0442 (18)	0.063 (2)	0.067 (2)	−0.0051 (16)	−0.0316 (17)	−0.0349 (19)

### Geometric parameters (Å, º)

**Table:**

Cl1—C1	1.743 (2)	C5—C6	1.389 (3)
Cl2—C3	1.729 (3)	C5—H5	0.9500
O1—C7	1.377 (3)	C6—H6	0.9500
O1—C4	1.397 (3)	C7—C8	1.386 (3)
O2—C13	1.198 (3)	C7—C12	1.394 (3)
O3—C13	1.331 (3)	C8—C9	1.383 (3)
O3—C14	1.454 (3)	C8—H8	0.9500
O4—N1	1.230 (3)	C9—C10	1.386 (3)
O5—N1	1.224 (3)	C9—H9	0.9500
N1—C10	1.462 (3)	C10—C11	1.393 (3)
C1—C6	1.379 (4)	C11—C12	1.383 (3)
C1—C2	1.381 (4)	C11—C13	1.504 (3)
C2—C3	1.386 (3)	C12—H12	0.9500
C2—H2	0.9500	C14—H14A	0.9800
C3—C4	1.382 (4)	C14—H14B	0.9800
C4—C5	1.381 (4)	C14—H14C	0.9800
			
C7—O1—C4	118.65 (19)	C8—C7—C12	121.1 (2)
C13—O3—C14	115.5 (2)	C9—C8—C7	119.1 (2)
O5—N1—O4	123.3 (2)	C9—C8—H8	120.5
O5—N1—C10	118.8 (2)	C7—C8—H8	120.5
O4—N1—C10	117.9 (2)	C8—C9—C10	119.6 (2)
C6—C1—C2	121.9 (2)	C8—C9—H9	120.2
C6—C1—Cl1	119.5 (2)	C10—C9—H9	120.2
C2—C1—Cl1	118.61 (19)	C9—C10—C11	121.9 (2)
C1—C2—C3	118.4 (2)	C9—C10—N1	117.7 (2)
C1—C2—H2	120.8	C11—C10—N1	120.3 (2)
C3—C2—H2	120.8	C12—C11—C10	118.1 (2)
C4—C3—C2	120.7 (2)	C12—C11—C13	117.5 (2)
C4—C3—Cl2	120.33 (19)	C10—C11—C13	124.3 (2)
C2—C3—Cl2	118.96 (19)	C11—C12—C7	120.2 (2)
C5—C4—C3	120.0 (2)	C11—C12—H12	119.9
C5—C4—O1	118.7 (2)	C7—C12—H12	119.9
C3—C4—O1	121.0 (2)	O2—C13—O3	124.4 (2)
C4—C5—C6	120.1 (2)	O2—C13—C11	124.5 (2)
C4—C5—H5	119.9	O3—C13—C11	111.0 (2)
C6—C5—H5	119.9	O3—C14—H14A	109.5
C1—C6—C5	118.8 (2)	O3—C14—H14B	109.5
C1—C6—H6	120.6	H14A—C14—H14B	109.5
C5—C6—H6	120.6	O3—C14—H14C	109.5
O1—C7—C8	124.2 (2)	H14A—C14—H14C	109.5
O1—C7—C12	114.7 (2)	H14B—C14—H14C	109.5
			
C6—C1—C2—C3	−0.2 (4)	C8—C9—C10—C11	−0.5 (4)
Cl1—C1—C2—C3	179.62 (19)	C8—C9—C10—N1	177.3 (2)
C1—C2—C3—C4	1.6 (4)	O5—N1—C10—C9	21.5 (3)
C1—C2—C3—Cl2	−177.51 (19)	O4—N1—C10—C9	−156.8 (2)
C2—C3—C4—C5	−2.1 (4)	O5—N1—C10—C11	−160.7 (2)
Cl2—C3—C4—C5	177.1 (2)	O4—N1—C10—C11	21.0 (3)
C2—C3—C4—O1	−175.6 (2)	C9—C10—C11—C12	1.2 (4)
Cl2—C3—C4—O1	3.6 (3)	N1—C10—C11—C12	−176.5 (2)
C7—O1—C4—C5	109.5 (3)	C9—C10—C11—C13	−174.7 (2)
C7—O1—C4—C3	−77.0 (3)	N1—C10—C11—C13	7.6 (4)
C3—C4—C5—C6	1.0 (4)	C10—C11—C12—C7	−1.1 (4)
O1—C4—C5—C6	174.6 (2)	C13—C11—C12—C7	175.1 (2)
C2—C1—C6—C5	−0.9 (4)	O1—C7—C12—C11	180.0 (2)
Cl1—C1—C6—C5	179.32 (19)	C8—C7—C12—C11	0.3 (4)
C4—C5—C6—C1	0.5 (4)	C14—O3—C13—O2	5.6 (4)
C4—O1—C7—C8	−8.8 (4)	C14—O3—C13—C11	−178.4 (2)
C4—O1—C7—C12	171.5 (2)	C12—C11—C13—O2	61.9 (4)
O1—C7—C8—C9	−179.2 (2)	C10—C11—C13—O2	−122.1 (3)
C12—C7—C8—C9	0.5 (4)	C12—C11—C13—O3	−114.1 (3)
C7—C8—C9—C10	−0.4 (4)	C10—C11—C13—O3	61.9 (3)

### Hydrogen-bond geometry (Å, º)

**Table:**

*D*—H···*A*	*D*—H	H···*A*	*D*···*A*	*D*—H···*A*
C5—H5···O4^i^	0.95	2.41	3.290 (3)	153
C6—H6···O5^ii^	0.95	2.43	3.243 (3)	143
C8—H8···O2^iii^	0.95	2.58	3.435 (3)	151

Symmetry codes: (i) −*x*+1, −*y*+1, −*z*+1; (ii) *x*, *y*, *z*+1; (iii) *x*+1, *y*, *z*.
